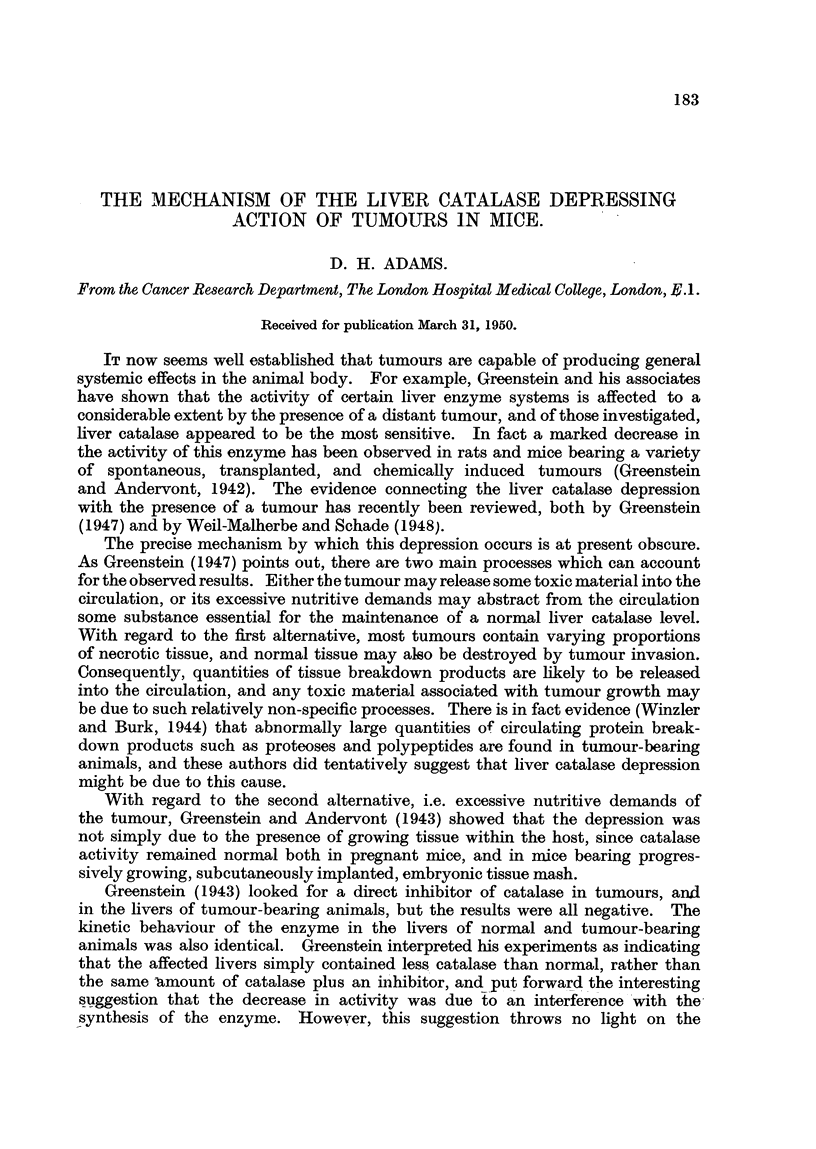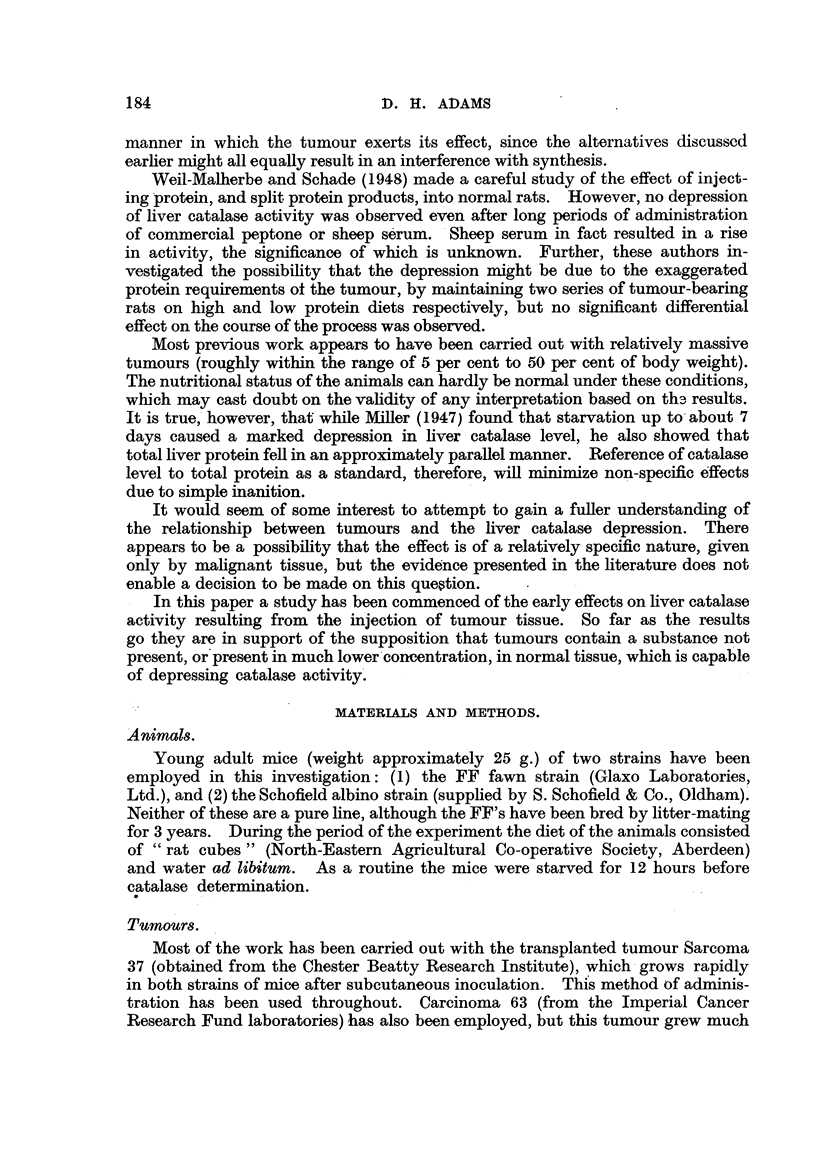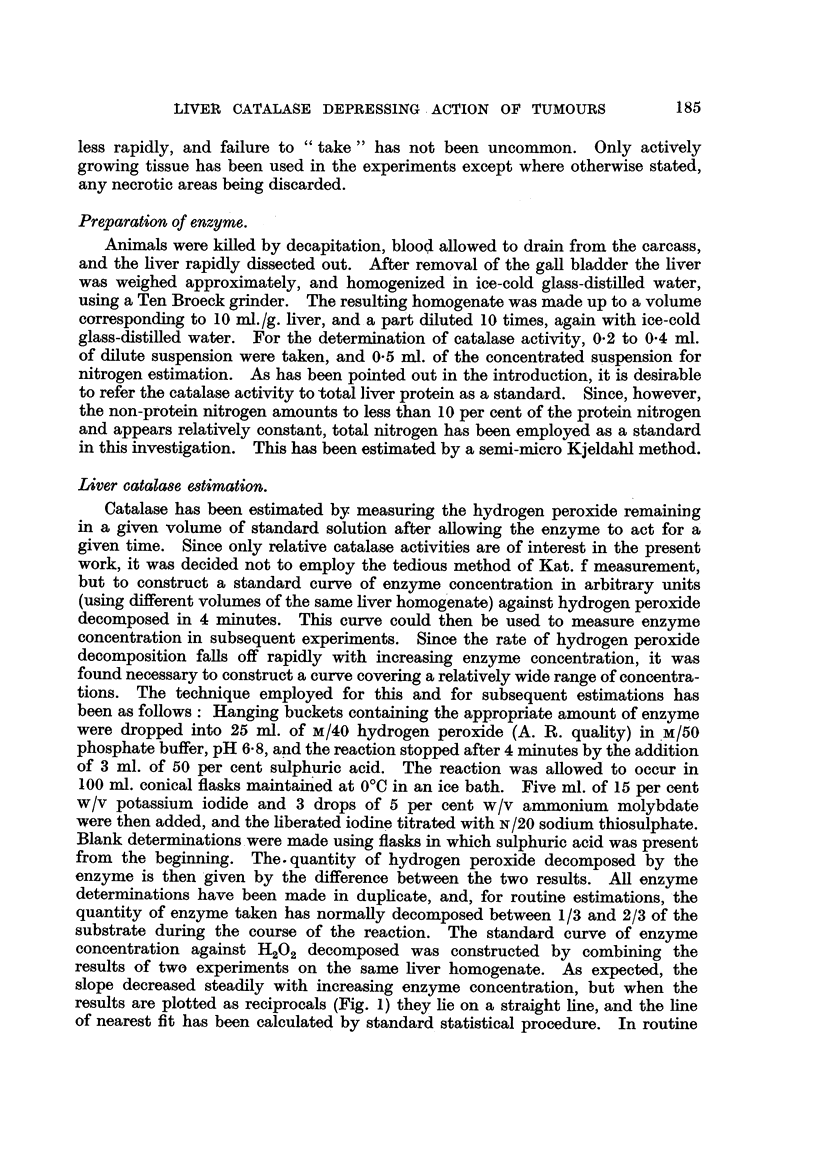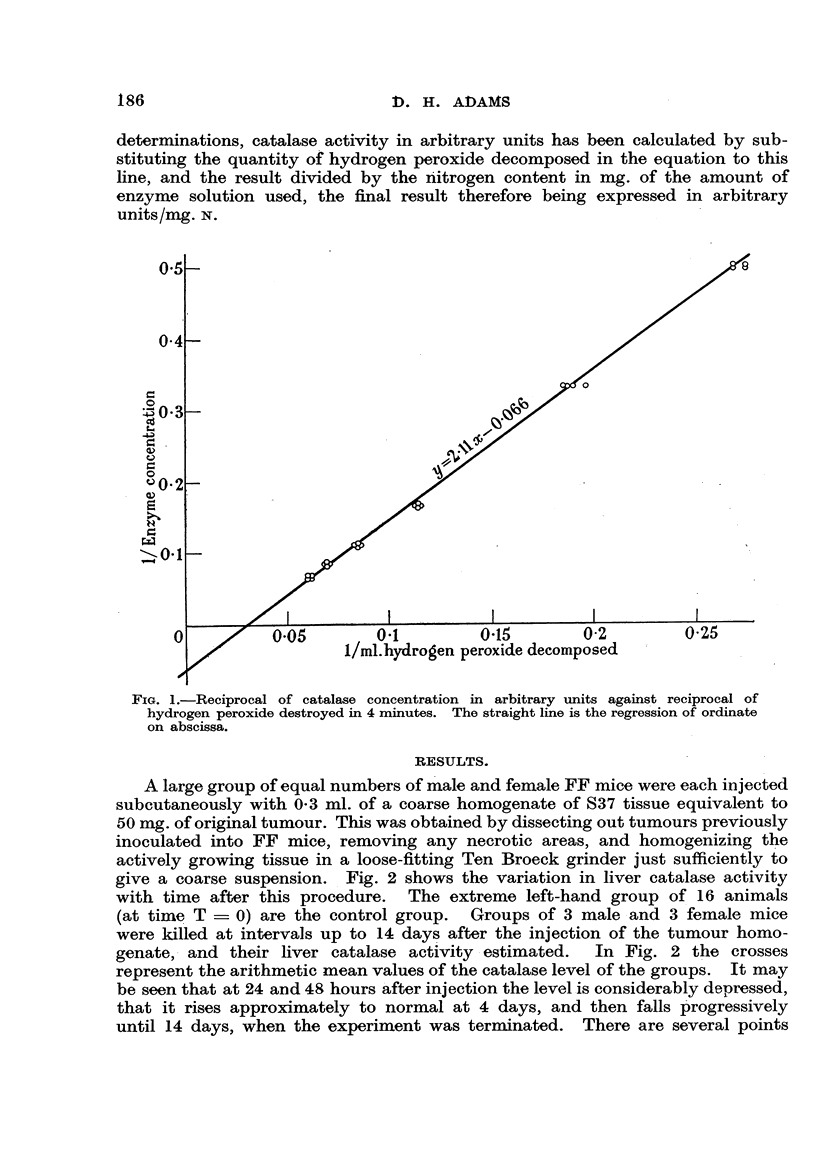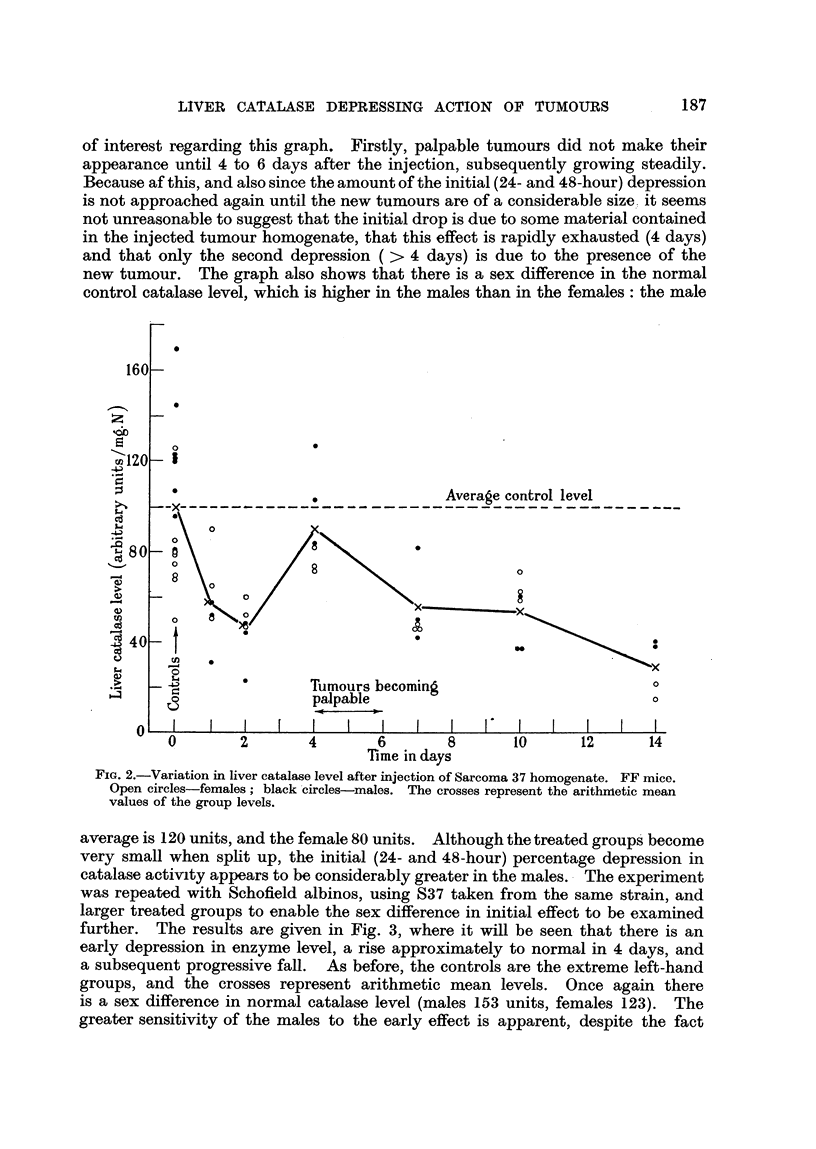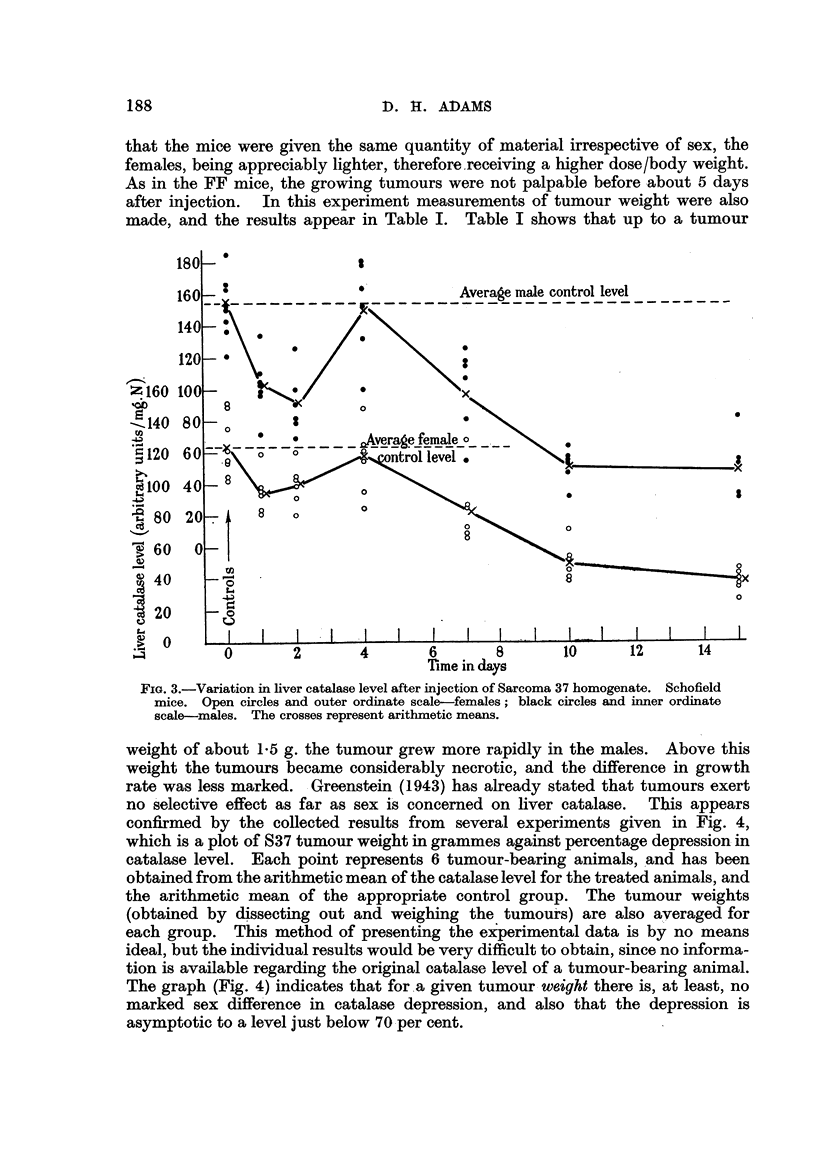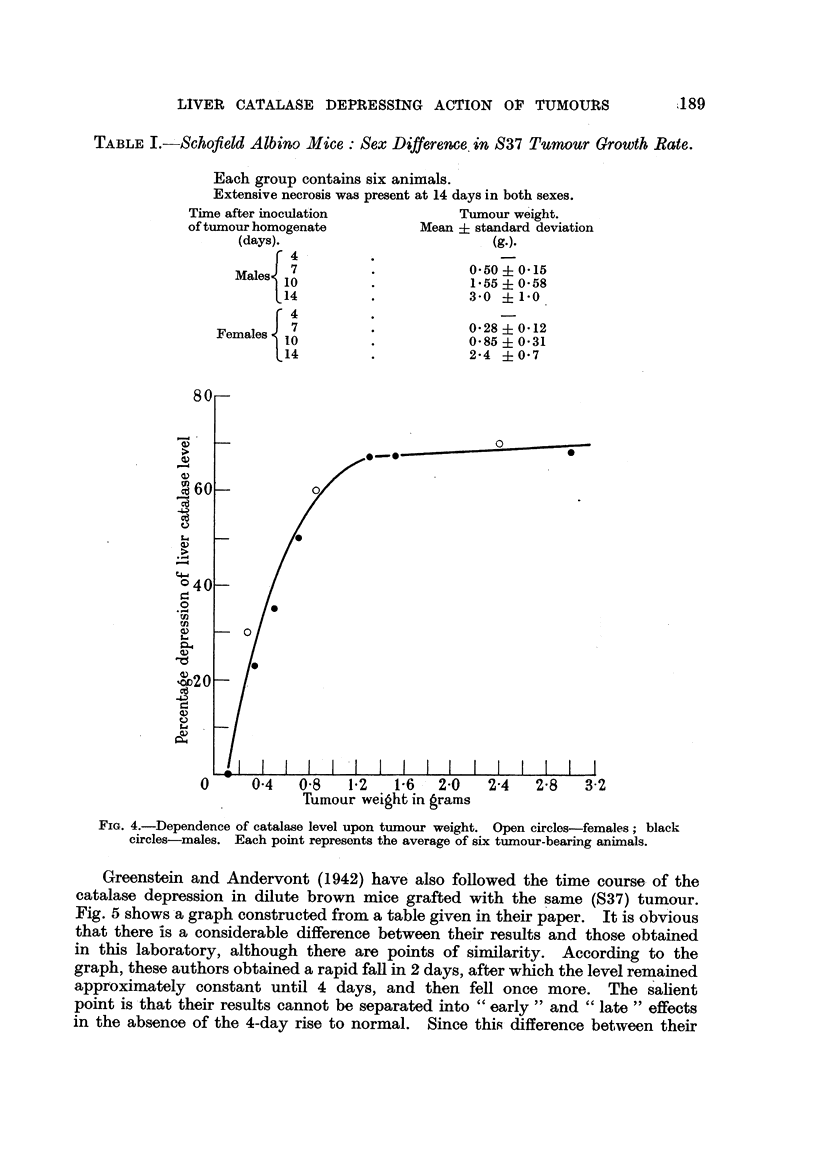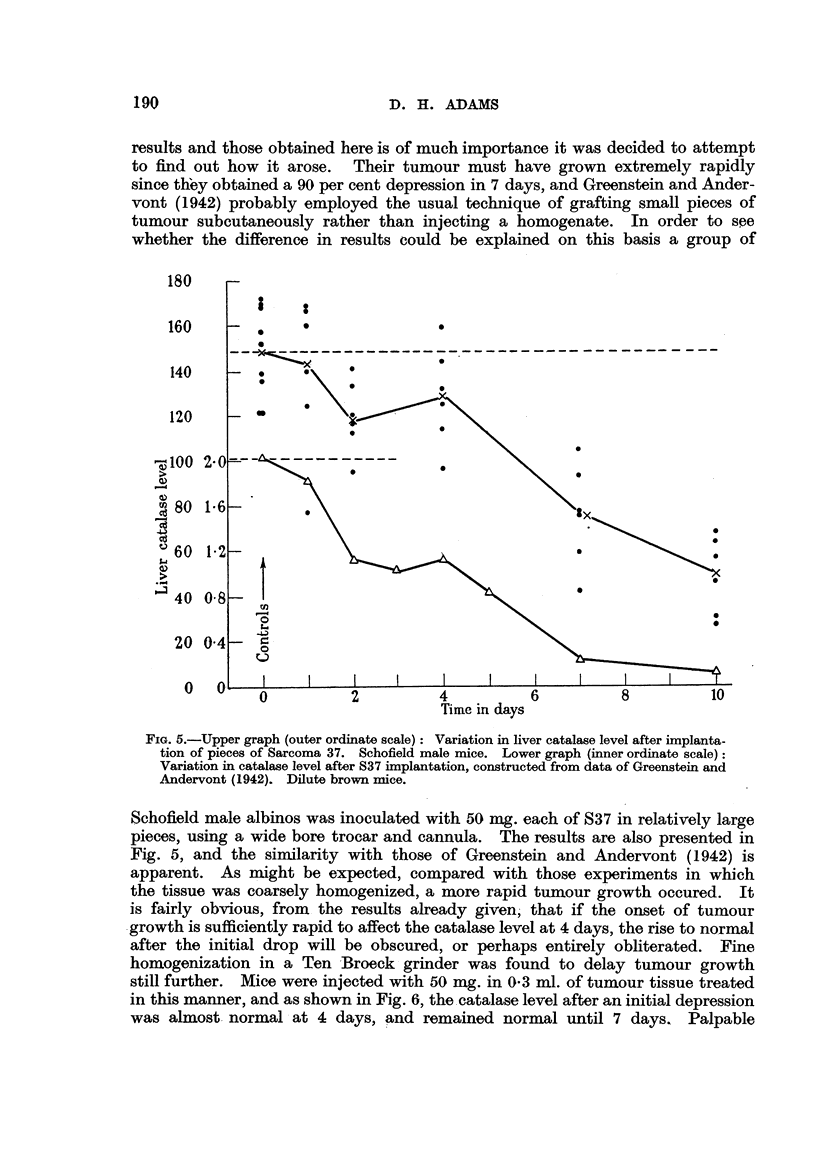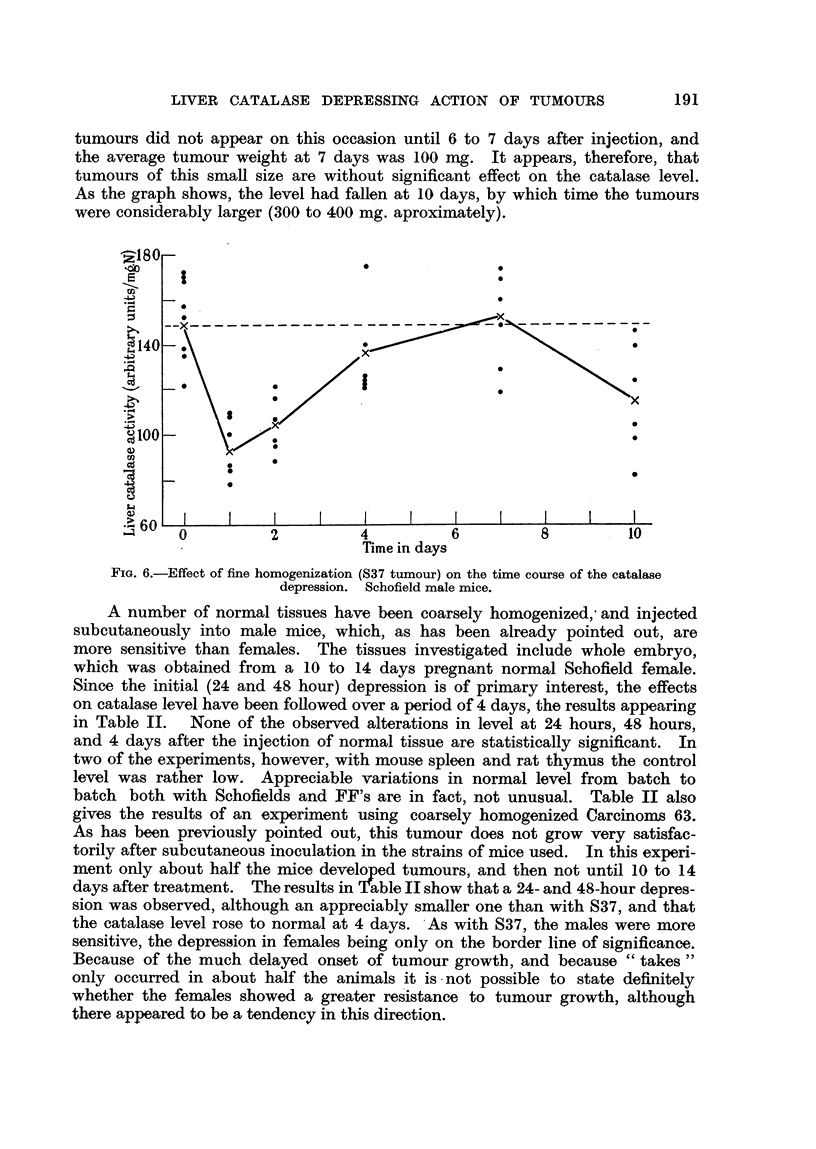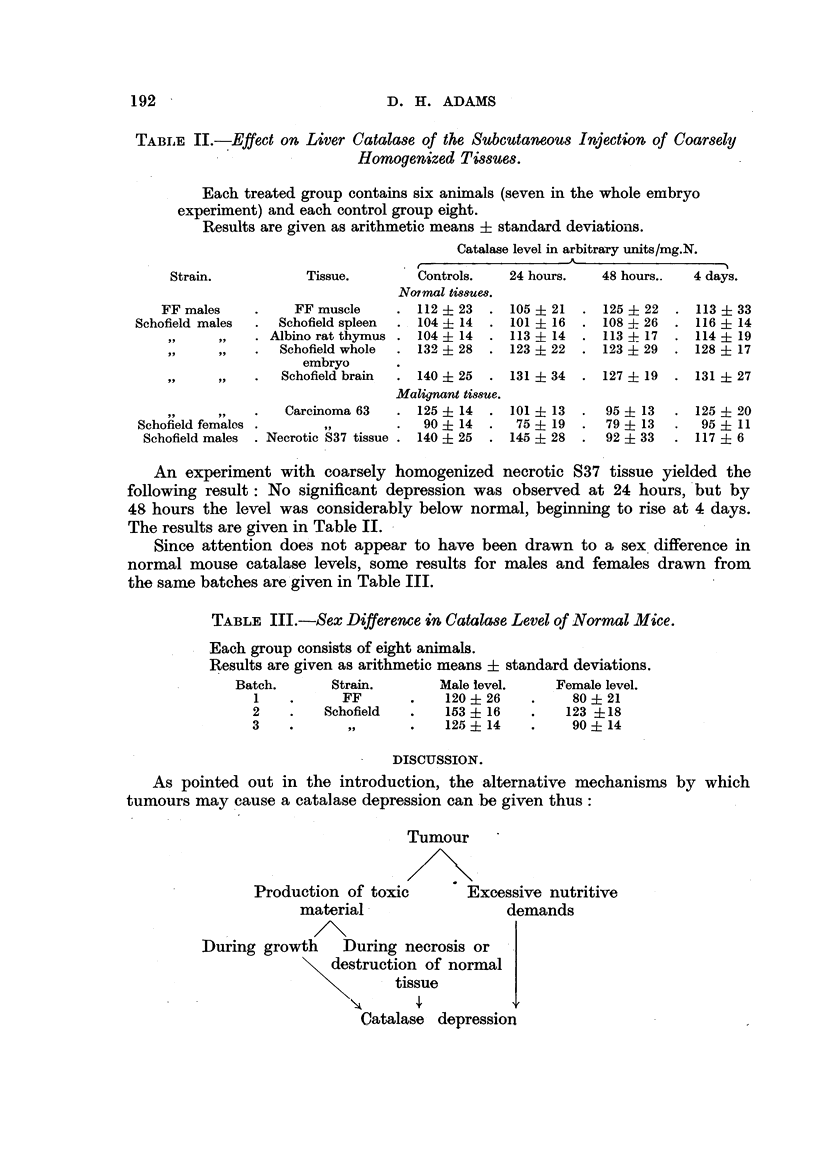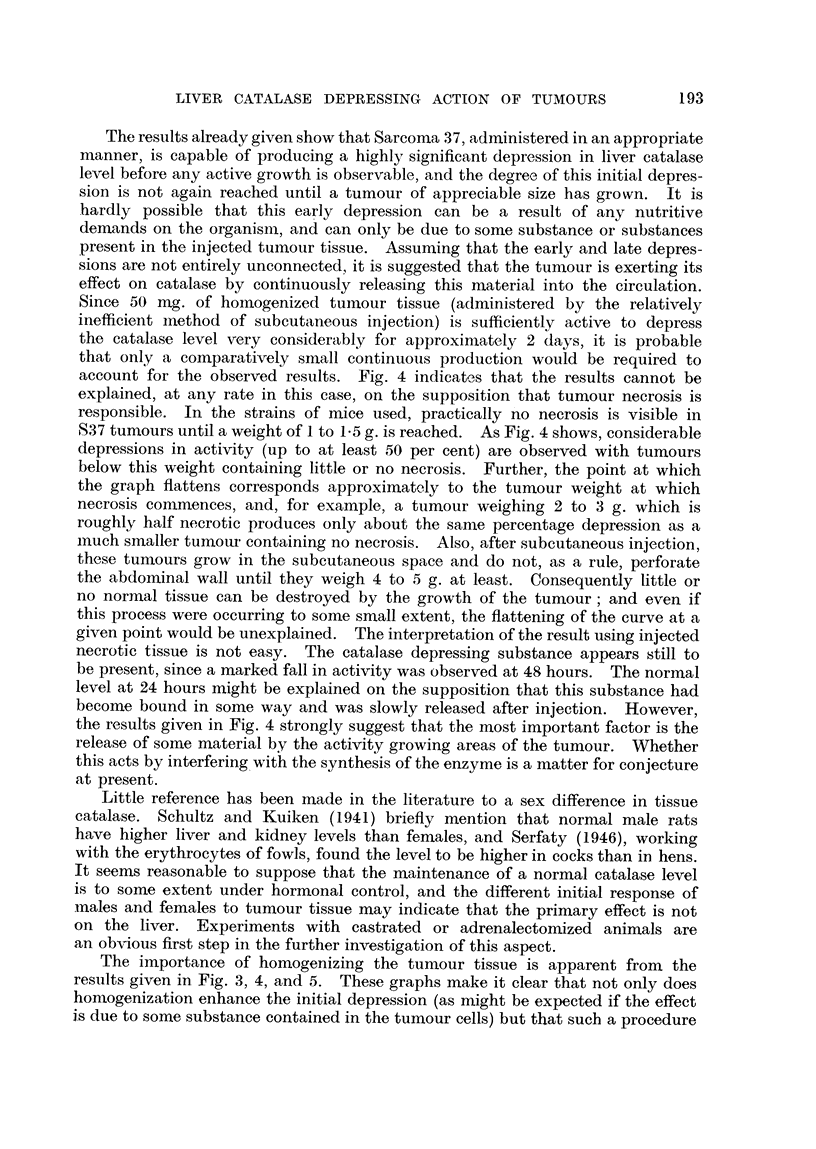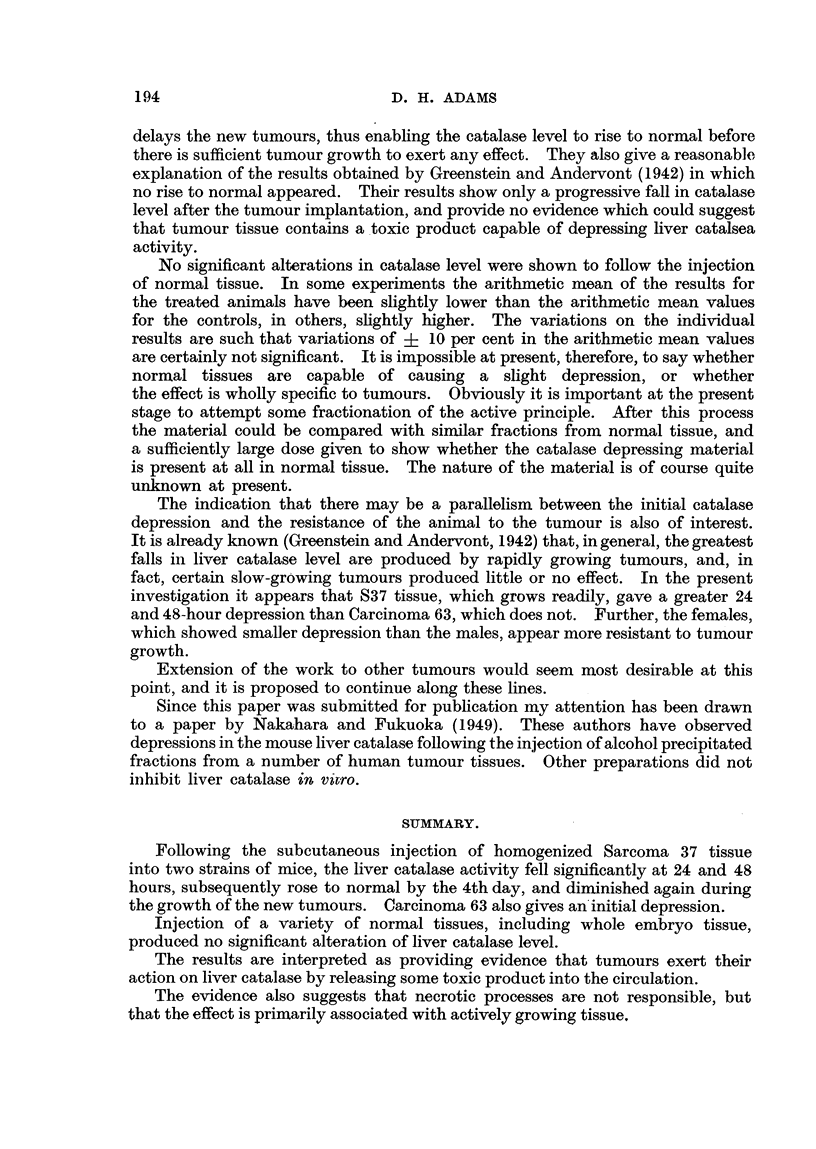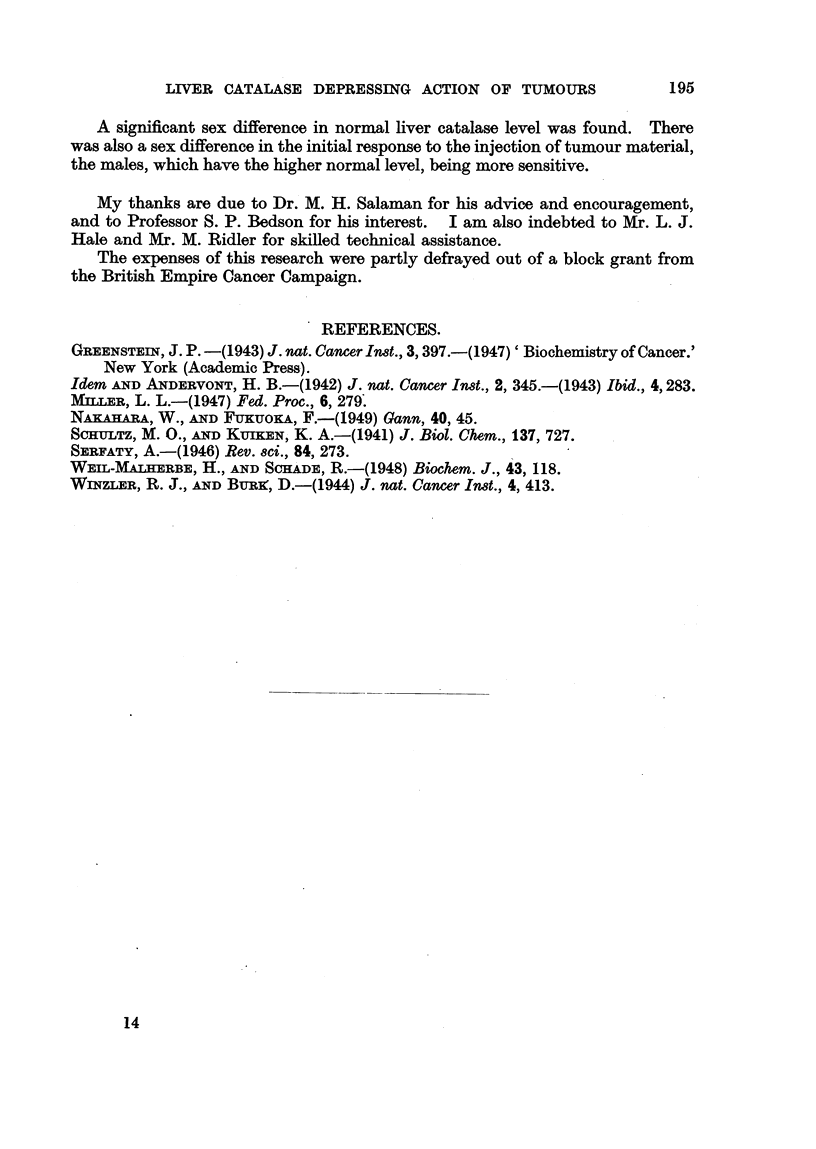# The Mechanism of the Liver Catalase Depressing Action of Tumours in Mice

**DOI:** 10.1038/bjc.1950.18

**Published:** 1950-06

**Authors:** D. H. Adams


### THE MECHANISM OF THE LIVER CATALASE DEPRESSING ACTION OF TUMOURS IN MICE.

D. H. ADAMS.

*From the Cancer Research Department, The London Hospital Medical College, London, E.1.*

Received for publication March 31, 1950.

It now seems well established that tumours are capable of producing general systemic effects in the animal body. For example, Greenstein and his associates have shown that the activity of certain liver enzyme systems is affected to a considerable extent by the presence of a distant tumour, and of those investigated, liver catalase appeared to be the most sensitive. In fact a marked decrease in the activity of this enzyme has been observed in rats and mice bearing a variety of spontaneous, transplanted, and chemically induced tumours (Greenstein and Andervont, 1942). The evidence connecting the liver catalase depression with the presence of a tumour has recently been reviewed, both by Greenstein (1947) and by Weil-Malherbe and Schade (1948).

The precise mechanism by which this depression occurs is at present obscure. As Greenstein (1947) points out, there are two main processes which can account for the observed results. Either the tumour may release some toxic material into the circulation, or its excessive nutritive demands may abstract from the circulation some substance essential for the maintenance of a normal liver catalase level. With regard to the first alternative, most tumours contain varying proportions of necrotic tissue, and normal tissue may also be destroyed by tumour invasion. Consequently, quantities of tissue breakdown products are likely to be released into the circulation, and any toxic material associated with tumour growth may be due to such relatively non-specific processes. There is in fact evidence (Winzler and Burk, 1944) that abnormally large quantities of circulating protein breakdown products such as proteoses and polypeptides are found in tumour-bearing animals, and these authors did tentatively suggest that liver catalase depression might be due to this cause.

With regard to the second alternative, i.e. excessive nutritive demands of the tumour, Greenstein and Andervont (1943) showed that the depression was not simply due to the presence of growing tissue within the host, since catalase activity remained normal both in pregnant mice, and in mice bearing progressively growing, subcutaneously implanted, embryonic tissue mash.

Greenstein (1943) looked for a direct inhibitor of catalase in tumours, and in the livers of tumour-bearing animals, but the results were all negative. The kinetic behaviour of the enzyme in the livers of normal and tumour-bearing animals was also identical. Greenstein interpreted his experiments as indicating that the affected livers simply contained less catalase than normal, rather than the same amount of catalase plus an inhibitor, and put forward the interesting suggestion that the decrease in activity was due to an interference with the synthesis of the enzyme. However, this suggestion throws no light on the manner in which the tumour exerts its effect, since the alternatives discussed earlier might all equally result in an interference with synthesis.

Weil-Malherbe and Schade (1948) made a careful study of the effect of injecting protein, and split protein products, into normal rats. However, no depression of liver catalase activity was observed even after long periods of administration of commercial peptone or sheep serum. Sheep serum in fact resulted in a rise in activity, the significance of which is unknown. Further, these authors investigated the possibility that the depression might be due to the exaggerated protein requirements ot the tumour, by maintaining two series of tumour-bearing rats on high and low protein diets respectively, but no significant differential effect on the course of the process was observed.

Most previous work appears to have been carried out with relatively massive tumours (roughly within the range of 5 per cent to 50 per cent of body weight). The nutritional status of the animals can hardly be normal under these conditions, which may cast doubt on the validity of any interpretation based on the results. It is true, however, that while Miller (1947) found that starvation up to about 7 days caused a marked depression in liver catalase level, he also showed that total liver protein fell in an approximately parallel manner. Reference of catalase level to total protein as a standard, therefore, will minimize non-specific effects due to simple inanition.

It would seem of some interest to attempt to gain a fuller understanding of the relationship between tumours and the liver catalase depression. There appears to be a possibility that the effect is of a relatively specific nature, given only by malignant tissue, but the evidence presented in the literature does not enable a decision to be made on this question.

In this paper a study has been commenced of the early effects on liver catalase activity resulting from the injection of tumour tissue. So far as the results go they are in support of the supposition that tumours contain a substance not present, or present in much lower concentration, in normal tissue, which is capable of depressing catalase activity.

### MATERIALS AND METHODS.

### *Animals.*

Young adult mice (weight approximately 25 g.) of two strains have been employed in this investigation: (1) the FF fawn strain (Glaxo Laboratories, Ltd.), and (2) the Schofield albino strain (supplied by S. Schofield & Co., Oldham). Neither of these are a pure line, although the FF's have been bred by litter-mating for 3 years. During the period of the experiment the diet of the animals consisted of "rat cubes" (North-Eastern Agricultural Co-operative Society, Aberdeen) and water *ad libitum*. As a routine the mice were starved for 12 hours before catalase determination.

### *Tumours.*

Most of the work has been carried out with the transplanted tumour Sarcoma 37 (obtained from the Chester Beatty Research Institute), which grows rapidly in both strains of mice after subcutaneous inoculation. This method of administration has been used throughout. Carcinoma 63 (from the Imperial Cancer Research Fund laboratories) has also been employed, but this tumour grew much less rapidly, and failure to " take " has not been uncommon. Only actively growing tissue has been used in the experiments except where otherwise stated, any necrotic areas being discarded.

*Preparation of enzyme.*

Animals were killed by decapitation, blood allowed to drain from the carcass, and the liver rapidly dissected out. After removal of the gall bladder the liver was weighed approximately, and homogenized in ice-cold glass-distilled water, using a Ten Broeck grinder. The resulting homogenate was made up to a volume corresponding to 10 ml./g. liver, and a part diluted 10 times, again with ice-cold glass-distilled water. For the determination of catalase activity, 0·2 to 0·4 ml. of dilute suspension were taken, and 0·5 ml. of the concentrated suspension for nitrogen estimation. As has been pointed out in the introduction, it is desirable to refer the catalase activity to total liver protein as a standard. Since, however, the non-protein nitrogen amounts to less than 10 per cent of the protein nitrogen and appears relatively constant, total nitrogen has been employed as a standard in this investigation. This has been estimated by a semi-micro Kjeldahl method.

*Liver catalase estimation.*

Catalase has been estimated by measuring the hydrogen peroxide remaining in a given volume of standard solution after allowing the enzyme to act for a given time. Since only relative catalase activities are of interest in the present work, it was decided not to employ the tedious method of Kat. f measurement, but to construct a standard curve of enzyme concentration in arbitrary units (using different volumes of the same liver homogenate) against hydrogen peroxide decomposed in 4 minutes. This curve could then be used to measure enzyme concentration in subsequent experiments. Since the rate of hydrogen peroxide decomposition falls off rapidly with increasing enzyme concentration, it was found necessary to construct a curve covering a relatively wide range of concentrations. The technique employed for this and for subsequent estimations has been as follows : Hanging buckets containing the appropriate amount of enzyme were dropped into 25 ml. of M/40 hydrogen peroxide (A. R. quality) in M/50 phosphate buffer, pH 6·8, and the reaction stopped after 4 minutes by the addition of 3 ml. of 50 per cent sulphuric acid. The reaction was allowed to occur in 100 ml. conical flasks maintained at 0°C in an ice bath. Five ml. of 15 per cent w/v potassium iodide and 3 drops of 5 per cent w/v ammonium molybdate were then added, and the liberated iodine titrated with N/20 sodium thiosulphate. Blank determinations were made using flasks in which sulphuric acid was present from the beginning. The quantity of hydrogen peroxide decomposed by the enzyme is then given by the difference between the two results. All enzyme determinations have been made in duplicate, and, for routine estimations, the quantity of enzyme taken has normally decomposed between 1/3 and 2/3 of the substrate during the course of the reaction. The standard curve of enzyme concentration against $H_2O_2$ decomposed was constructed by combining the results of two experiments on the same liver homogenate. As expected, the slope decreased steadily with increasing enzyme concentration, but when the results are plotted as reciprocals (Fig. 1) they lie on a straight line, and the line of nearest fit has been calculated by standard statistical procedure. In routine determinations, catalase activity in arbitrary units has been calculated by substituting the quantity of hydrogen peroxide decomposed in the equation to this line, and the result divided by the nitrogen content in mg. of the amount of enzyme solution used, the final result therefore being expressed in arbitrary units/mg. N.

FIG. 1.—Reciprocal of catalase concentration in arbitrary units against reciprocal of hydrogen peroxide destroyed in 4 minutes. The straight line is the regression of ordinate on abscissa.

### RESULTS.

A large group of equal numbers of male and female FF mice were each injected subcutaneously with 0·3 ml. of a coarse homogenate of S37 tissue equivalent to 50 mg. of original tumour. This was obtained by dissecting out tumours previously inoculated into FF mice, removing any necrotic areas, and homogenizing the actively growing tissue in a loose-fitting Ten Broeck grinder just sufficiently to give a coarse suspension. Fig. 2 shows the variation in liver catalase activity with time after this procedure. The extreme left-hand group of 16 animals (at time T = 0) are the control group. Groups of 3 male and 3 female mice were killed at intervals up to 14 days after the injection of the tumour homogenate, and their liver catalase activity estimated. In Fig. 2 the crosses represent the arithmetic mean values of the catalase level of the groups. It may be seen that at 24 and 48 hours after injection the level is considerably depressed, that it rises approximately to normal at 4 days, and then falls progressively until 14 days, when the experiment was terminated. There are several points of interest regarding this graph. Firstly, palpable tumours did not make their appearance until 4 to 6 days after the injection, subsequently growing steadily. Because af this, and also since the amount of the initial (24- and 48-hour) depression is not approached again until the new tumours are of a considerable size, it seems not unreasonable to suggest that the initial drop is due to some material contained in the injected tumour homogenate, that this effect is rapidly exhausted (4 days) and that only the second depression ( > 4 days) is due to the presence of the new tumour. The graph also shows that there is a sex difference in the normal control catalase level, which is higher in the males than in the females : the male

FIG. 2.—Variation in liver catalase level after injection of Sarcoma 37 homogenate. FF mice. Open circles—females ; black circles—males. The crosses represent the arithmetic mean values of the group levels.

average is 120 units, and the female 80 units. Although the treated groups become very small when split up, the initial (24- and 48-hour) percentage depression in catalase activity appears to be considerably greater in the males. The experiment was repeated with Schofield albinos, using S37 taken from the same strain, and larger treated groups to enable the sex difference in initial effect to be examined further. The results are given in Fig. 3, where it will be seen that there is an early depression in enzyme level, a rise approximately to normal in 4 days, and a subsequent progressive fall. As before, the controls are the extreme left-hand groups, and the crosses represent arithmetic mean levels. Once again there is a sex difference in normal catalase level (males 153 units, females 123). The greater sensitivity of the males to the early effect is apparent, despite the fact that the mice were given the same quantity of material irrespective of sex, the females, being appreciably lighter, therefore receiving a higher dose/body weight. As in the FF mice, the growing tumours were not palpable before about 5 days after injection. In this experiment measurements of tumour weight were also made, and the results appear in Table I. Table I shows that up to a tumour

FIG. 3.—Variation in liver catalase level after injection of Sarcoma 37 homogenate. Schofield mice. Open circles and outer ordinate scale—females; black circles and inner ordinate scale—males. The crosses represent arithmetic means.

weight of about 1·5 g. the tumour grew more rapidly in the males. Above this weight the tumours became considerably necrotic, and the difference in growth rate was less marked. Greenstein (1943) has already stated that tumours exert no selective effect as far as sex is concerned on liver catalase. This appears confirmed by the collected results from several experiments given in Fig. 4, which is a plot of S37 tumour weight in grammes against percentage depression in catalase level. Each point represents 6 tumour-bearing animals, and has been obtained from the arithmetic mean of the catalase level for the treated animals, and the arithmetic mean of the appropriate control group. The tumour weights (obtained by dissecting out and weighing the tumours) are also averaged for each group. This method of presenting the experimental data is by no means ideal, but the individual results would be very difficult to obtain, since no information is available regarding the original catalase level of a tumour-bearing animal. The graph (Fig. 4) indicates that for a given tumour *weight* there is, at least, no marked sex difference in catalase depression, and also that the depression is asymptotic to a level just below 70 per cent.

TABLE I.—*Schofield Albino Mice : Sex Difference in S37 Tumour Growth Rate.*

Each group contains six animals.
Extensive necrosis was present at 14 days in both sexes.

| | Time after inoculation of tumour homogenate (days). | Tumour weight. Mean ± standard deviation (g.). |
|---|---|---|
| Males | 4 | — |
| | 7 | 0·50 ± 0·15 |
| | 10 | 1·55 ± 0·58 |
| | 14 | 3·0 ± 1·0 |
| Females | 4 | — |
| | 7 | 0·28 ± 0·12 |
| | 10 | 0·85 ± 0·31 |
| | 14 | 2·4 ± 0·7 |

FIG. 4.—Dependence of catalase level upon tumour weight. Open circles—females ; black circles—males. Each point represents the average of six tumour-bearing animals.

Greenstein and Andervont (1942) have also followed the time course of the catalase depression in dilute brown mice grafted with the same (S37) tumour. Fig. 5 shows a graph constructed from a table given in their paper. It is obvious that there is a considerable difference between their results and those obtained in this laboratory, although there are points of similarity. According to the graph, these authors obtained a rapid fall in 2 days, after which the level remained approximately constant until 4 days, and then fell once more. The salient point is that their results cannot be separated into " early " and " late " effects in the absence of the 4-day rise to normal. Since this difference between their results and those obtained here is of much importance it was decided to attempt to find out how it arose. Their tumour must have grown extremely rapidly since they obtained a 90 per cent depression in 7 days, and Greenstein and Andervont (1942) probably employed the usual technique of grafting small pieces of tumour subcutaneously rather than injecting a homogenate. In order to see whether the difference in results could be explained on this basis a group of

Fig. 5.—Upper graph (outer ordinate scale) : Variation in liver catalase level after implantation of pieces of Sarcoma 37. Schofield male mice. Lower graph (inner ordinate scale) : Variation in catalase level after S37 implantation, constructed from data of Greenstein and Andervont (1942). Dilute brown mice.

Schofield male albinos was inoculated with 50 mg. each of S37 in relatively large pieces, using a wide bore trocar and cannula. The results are also presented in Fig. 5, and the similarity with those of Greenstein and Andervont (1942) is apparent. As might be expected, compared with those experiments in which the tissue was coarsely homogenized, a more rapid tumour growth occured. It is fairly obvious, from the results already given, that if the onset of tumour growth is sufficiently rapid to affect the catalase level at 4 days, the rise to normal after the initial drop will be obscured, or perhaps entirely obliterated. Fine homogenization in a Ten Broeck grinder was found to delay tumour growth still further. Mice were injected with 50 mg. in 0·3 ml. of tumour tissue treated in this manner, and as shown in Fig. 6, the catalase level after an initial depression was almost normal at 4 days, and remained normal until 7 days. Palpable tumours did not appear on this occasion until 6 to 7 days after injection, and the average tumour weight at 7 days was 100 mg. It appears, therefore, that tumours of this small size are without significant effect on the catalase level. As the graph shows, the level had fallen at 10 days, by which time the tumours were considerably larger (300 to 400 mg. aproximately).

FIG. 6.—Effect of fine homogenization (S37 tumour) on the time course of the catalase depression. Schofield male mice.

A number of normal tissues have been coarsely homogenized, and injected subcutaneously into male mice, which, as has been already pointed out, are more sensitive than females. The tissues investigated include whole embryo, which was obtained from a 10 to 14 days pregnant normal Schofield female. Since the initial (24 and 48 hour) depression is of primary interest, the effects on catalase level have been followed over a period of 4 days, the results appearing in Table II. None of the observed alterations in level at 24 hours, 48 hours, and 4 days after the injection of normal tissue are statistically significant. In two of the experiments, however, with mouse spleen and rat thymus the control level was rather low. Appreciable variations in normal level from batch to batch both with Schofields and FF's are in fact, not unusual. Table II also gives the results of an experiment using coarsely homogenized Carcinoms 63. As has been previously pointed out, this tumour does not grow very satisfactorily after subcutaneous inoculation in the strains of mice used. In this experiment only about half the mice developed tumours, and then not until 10 to 14 days after treatment. The results in Table II show that a 24- and 48-hour depression was observed, although an appreciably smaller one than with S37, and that the catalase level rose to normal at 4 days. As with S37, the males were more sensitive, the depression in females being only on the border line of significance. Because of the much delayed onset of tumour growth, and because " takes " only occurred in about half the animals it is not possible to state definitely whether the females showed a greater resistance to tumour growth, although there appeared to be a tendency in this direction.

TABLE II.—*Effect on Liver Catalase of the Subcutaneous Injection of Coarsely Homogenized Tissues.*

Each treated group contains six animals (seven in the whole embryo experiment) and each control group eight.
Results are given as arithmetic means ± standard deviations.

| | | Catalase level in arbitrary units/mg.N. | | | |
|---|---|---|---|---|---|
| Strain. | Tissue. | Controls. | 24 hours. | 48 hours.. | 4 days. |
| | | *Normal tissues.* | | | |
| FF males | FF muscle | 112 ± 23 | 105 ± 21 | 125 ± 22 | 113 ± 33 |
| Schofield males | Schofield spleen | 104 ± 14 | 101 ± 16 | 108 ± 26 | 116 ± 14 |
| „ „ | Albino rat thymus | 104 ± 14 | 113 ± 14 | 113 ± 17 | 114 ± 19 |
| „ „ | Schofield whole embryo | 132 ± 28 | 123 ± 22 | 123 ± 29 | 128 ± 17 |
| „ „ | Schofield brain | 140 ± 25 | 131 ± 34 | 127 ± 19 | 131 ± 27 |
| | | *Malignant tissue.* | | | |
| „ „ | Carcinoma 63 | 125 ± 14 | 101 ± 13 | 95 ± 13 | 125 ± 20 |
| Schofield femalos | „ | 90 ± 14 | 75 ± 19 | 79 ± 13 | 95 ± 11 |
| Schofield males | Necrotic S37 tissue | 140 ± 25 | 145 ± 28 | 92 ± 33 | 117 ± 6 |

An experiment with coarsely homogenized necrotic S37 tissue yielded the following result : No significant depression was observed at 24 hours, but by 48 hours the level was considerably below normal, beginning to rise at 4 days. The results are given in Table II.

Since attention does not appear to have been drawn to a sex difference in normal mouse catalase levels, some results for males and females drawn from the same batches are given in Table III.

TABLE III.—*Sex Difference in Catalase Level of Normal Mice.*

Each group consists of eight animals.
Results are given as arithmetic means ± standard deviations.

| Batch. | Strain. | Male level. | Female level. |
|---|---|---|---|
| 1 | FF | 120 ± 26 | 80 ± 21 |
| 2 | Schofield | 153 ± 16 | 123 ±18 |
| 3 | „ | 125 ± 14 | 90 ± 14 |

DISCUSSION.

As pointed out in the introduction, the alternative mechanisms by which tumours may cause a catalase depression can be given thus :

The results already given show that Sarcoma 37, administered in an appropriate manner, is capable of producing a highly significant depression in liver catalase level before any active growth is observable, and the degree of this initial depression is not again reached until a tumour of appreciable size has grown. It is hardly possible that this early depression can be a result of any nutritive demands on the organism, and can only be due to some substance or substances present in the injected tumour tissue. Assuming that the early and late depressions are not entirely unconnected, it is suggested that the tumour is exerting its effect on catalase by continuously releasing this material into the circulation. Since 50 mg. of homogenized tumour tissue (administered by the relatively inefficient method of subcutaneous injection) is sufficiently active to depress the catalase level very considerably for approximately 2 days, it is probable that only a comparatively small continuous production would be required to account for the observed results. Fig. 4 indicates that the results cannot be explained, at any rate in this case, on the supposition that tumour necrosis is responsible. In the strains of mice used, practically no necrosis is visible in S37 tumours until a weight of 1 to 1·5 g. is reached. As Fig. 4 shows, considerable depressions in activity (up to at least 50 per cent) are observed with tumours below this weight containing little or no necrosis. Further, the point at which the graph flattens corresponds approximately to the tumour weight at which necrosis commences, and, for example, a tumour weighing 2 to 3 g. which is roughly half necrotic produces only about the same percentage depression as a much smaller tumour containing no necrosis. Also, after subcutaneous injection, these tumours grow in the subcutaneous space and do not, as a rule, perforate the abdominal wall until they weigh 4 to 5 g. at least. Consequently little or no normal tissue can be destroyed by the growth of the tumour ; and even if this process were occurring to some small extent, the flattening of the curve at a given point would be unexplained. The interpretation of the result using injected necrotic tissue is not easy. The catalase depressing substance appears still to be present, since a marked fall in activity was observed at 48 hours. The normal level at 24 hours might be explained on the supposition that this substance had become bound in some way and was slowly released after injection. However, the results given in Fig. 4 strongly suggest that the most important factor is the release of some material by the activity growing areas of the tumour. Whether this acts by interfering with the synthesis of the enzyme is a matter for conjecture at present.

Little reference has been made in the literature to a sex difference in tissue catalase. Schultz and Kuiken (1941) briefly mention that normal male rats have higher liver and kidney levels than females, and Serfaty (1946), working with the erythrocytes of fowls, found the level to be higher in cocks than in hens. It seems reasonable to suppose that the maintenance of a normal catalase level is to some extent under hormonal control, and the different initial response of males and females to tumour tissue may indicate that the primary effect is not on the liver. Experiments with castrated or adrenalectomized animals are an obvious first step in the further investigation of this aspect.

The importance of homogenizing the tumour tissue is apparent from the results given in Fig. 3, 4, and 5. These graphs make it clear that not only does homogenization enhance the initial depression (as might be expected if the effect is due to some substance contained in the tumour cells) but that such a procedure delays the new tumours, thus enabling the catalase level to rise to normal before there is sufficient tumour growth to exert any effect. They also give a reasonable explanation of the results obtained by Greenstein and Andervont (1942) in which no rise to normal appeared. Their results show only a progressive fall in catalase level after the tumour implantation, and provide no evidence which could suggest that tumour tissue contains a toxic product capable of depressing liver catalsea activity.

No significant alterations in catalase level were shown to follow the injection of normal tissue. In some experiments the arithmetic mean of the results for the treated animals have been slightly lower than the arithmetic mean values for the controls, in others, slightly higher. The variations on the individual results are such that variations of $\pm$ 10 per cent in the arithmetic mean values are certainly not significant. It is impossible at present, therefore, to say whether normal tissues are capable of causing a slight depression, or whether the effect is wholly specific to tumours. Obviously it is important at the present stage to attempt some fractionation of the active principle. After this process the material could be compared with similar fractions from normal tissue, and a sufficiently large dose given to show whether the catalase depressing material is present at all in normal tissue. The nature of the material is of course quite unknown at present.

The indication that there may be a parallelism between the initial catalase depression and the resistance of the animal to the tumour is also of interest. It is already known (Greenstein and Andervont, 1942) that, in general, the greatest falls in liver catalase level are produced by rapidly growing tumours, and, in fact, certain slow-growing tumours produced little or no effect. In the present investigation it appears that S37 tissue, which grows readily, gave a greater 24 and 48-hour depression than Carcinoma 63, which does not. Further, the females, which showed smaller depression than the males, appear more resistant to tumour growth.

Extension of the work to other tumours would seem most desirable at this point, and it is proposed to continue along these lines.

Since this paper was submitted for publication my attention has been drawn to a paper by Nakahara and Fukuoka (1949). These authors have observed depressions in the mouse liver catalase following the injection of alcohol precipitated fractions from a number of human tumour tissues. Other preparations did not inhibit liver catalase *in viiro.*

### SUMMARY.

Following the subcutaneous injection of homogenized Sarcoma 37 tissue into two strains of mice, the liver catalase activity fell significantly at 24 and 48 hours, subsequently rose to normal by the 4th day, and diminished again during the growth of the new tumours. Carcinoma 63 also gives an initial depression.

Injection of a variety of normal tissues, including whole embryo tissue, produced no significant alteration of liver catalase level.

The results are interpreted as providing evidence that tumours exert their action on liver catalase by releasing some toxic product into the circulation.

The evidence also suggests that necrotic processes are not responsible, but that the effect is primarily associated with actively growing tissue.

A significant sex difference in normal liver catalase level was found. There was also a sex difference in the initial response to the injection of tumour material, the males, which have the higher normal level, being more sensitive.

My thanks are due to Dr. M. H. Salaman for his advice and encouragement, and to Professor S. P. Bedson for his interest. I am also indebted to Mr. L. J. Hale and Mr. M. Ridler for skilled technical assistance.

The expenses of this research were partly defrayed out of a block grant from the British Empire Cancer Campaign.

### REFERENCES.

Greenstein, J. P.—(1943) *J. nat. Cancer Inst.*, **3**, 397.—(1947) 'Biochemistry of Cancer.' New York (Academic Press).

*Idem* and Andervont, H. B.—(1942) *J. nat. Cancer Inst.*, **2**, 345.—(1943) *Ibid.*, **4**, 283.

Miller, L. L.—(1947) *Fed. Proc.*, **6**, 279.

Nakahara, W., and Fukuoka, F.—(1949) *Gann*, **40**, 45.

Schultz, M. O., and Kuiken, K. A.—(1941) *J. Biol. Chem.*, **137**, 727.

Serfaty, A.—(1946) *Rev. sci.*, **84**, 273.

Weil-Malherbe, H., and Schade, R.—(1948) *Biochem. J.*, **43**, 118.

Winzler, R. J., and Burk, D.—(1944) *J. nat. Cancer Inst.*, **4**, 413.